# Modified C-H flap for simultaneous nipple reconstruction during autologous breast reconstruction: Surgical tips for safety and cosmesis

**DOI:** 10.1097/MD.0000000000012460

**Published:** 2018-09-21

**Authors:** Jung Soo Yoon, Jung Woo Chang, Hee Chang Ahn, Min Sung Chung

**Affiliations:** a Department of Plastic and Reconstructive Surgery, Hanyang University Medical Center, Hanyang University College of Medicine, Seoul; b Department of Plastic and Reconstructive Surgery, Hanyang University Guri Hospital, Hanyang University College of Medicine, Guri; c Department of Surgery, Hanyang University Medical Center, Hanyang University College of Medicine, Seoul, Korea.

**Keywords:** breast reconstruction, C-H flap, nipple reconstruction, one stage reconstruction

## Abstract

**Background:**

Reconstruction of the nipple–areolar complex is the final stage of breast reconstruction. Nipple reconstruction is usually performed several months after breast reconstruction, because simultaneous reconstruction is thought to be risky. Here, we introduce our experiences of 1-stage procedures with immediate reconstruction of the nipple–areolar complex during autologous breast reconstruction.

**Methods:**

Between 2008 and 2015, 51 mastectomy patients underwent 1-stage breast and nipple reconstruction. All cases were reconstructed immediately with autologous tissue for the breast mound. The patients were divided into 2 groups according to the method of nipple–areolar complex reconstruction. In group A, 23 cases were reconstructed with a classical C-H flap, also known as the Hammond flap. In group B, 28 cases were reconstructed with a modified C-H flap, which is the evolved form of the classical Hammond flap. The nipple–areolar complex was evaluated preoperatively, immediately postoperatively, and 1 year postoperatively. Postoperative complications were also evaluated.

**Results:**

The mean projection of the reconstructed nipple decreased by approximately 50% in group A and 38% in group B during the postoperative 1 year. However, the reconstructed nipple width and areolar diameter did not show a significant change in either group. Group A showed 26% of complication rate and 17% of revision rate, whereas group B showed 11% of complication rate and 4% of revision rate. However, no major complications such as complete necrosis of the reconstructed nipple, were observed in any patients.

**Conclusion:**

The modified technique group showed superior results in terms of safety and cosmesis. With our modified C-H flap method, simultaneous breast and nipple reconstruction is safe and has satisfactory results.

## 1 Introduction

Since autologous breast reconstruction was introduced in 1906 by Ombredanne,^[1]^ it has been evolved and widely used for breast mound reconstruction in mastectomy patients. For complete breast reconstruction, creating a nipple–areolar complex is essential, and such a technique was introduced in 1940s by Adams for the first time.^[2,3]^ Making the nipple–areolar complex is the final stage of breast reconstruction,^[4–8]^ and diverse techniques have evolved.^[9–12]^

This is an essential step of the reconstruction in mastectomy patients, but many reconstructive surgeons still avoid simultaneous nipple–areolar complex reconstruction during breast reconstruction. There are some reasons for this avoidance. Simultaneous reconstruction can result in circulatory problems such as partial or complete necrosis of the reconstructed nipple, and it leaves the possibility of a revisional operation. Further, unfavorable aesthetic results in long-term follow-up, such as a loss of projection or width in the reconstructed nipple, are another reason for avoiding simultaneous reconstruction.^[13–17]^

To overcome these risks, we have developed a safe method for simultaneous nipple–areolar complex reconstruction during breast reconstruction. The purpose of this study is to introduce this modified technique, which we found to yield better results in simultaneous 1-stage nipple and breast mound reconstruction in mastectomy patients.

## 2 Methods

This study was conducted in conformity with the World Medical Association Declaration of Helsinki, and the protocol was approved by the Institutional Review Board of Hanyang University Medical Center on November 14, 2017 (HYUH-2017-11-004). From March 2008 to January 2015, 51 mastectomy patients underwent immediate autologous breast reconstruction and simultaneous nipple–areolar complex reconstruction. The mean age of the patients was 47.2 years (range: 22–64 years) and their mean body-mass index was 23.6 kg/m^2^. The average follow-up period was 21.3 months.

All patients underwent a skin-sparing mastectomy performed by a single breast surgeon. In all cases, skin incisions were done from the nipple to the axilla, and the breast tissue and the nipple–areolar complex were completely resected. All the breast mound reconstructions were performed with autologous flaps such as a muscle sparing free transverse rectus abdominis musculocutaneous (TRAM) flap (23 cases), a pedicled TRAM flap (22 cases), and a pedicled latissimus dorsi (LD) flap (6 cases). The reconstructions were performed by a single plastic surgeon.

The patients were divided into 2 groups according to the method of nipple–areolar complex reconstruction. Among the 51 cases, group A (23 cases) received a classical C-H flap for nipple–areolar reconstruction, whereas group B (28 cases) received a modified C-H flap for nipple–areolar reconstruction. The classical C-H flap is a method also known as the Hammond flap, and it is a modified form of the C-V flap. When designing the flap, a blunt-tip flap was preferred to a V-shaped flap. After elevating the flap, the reconstructed nipple was finished by wound closure with a C-H shape.

**Figure 1:**
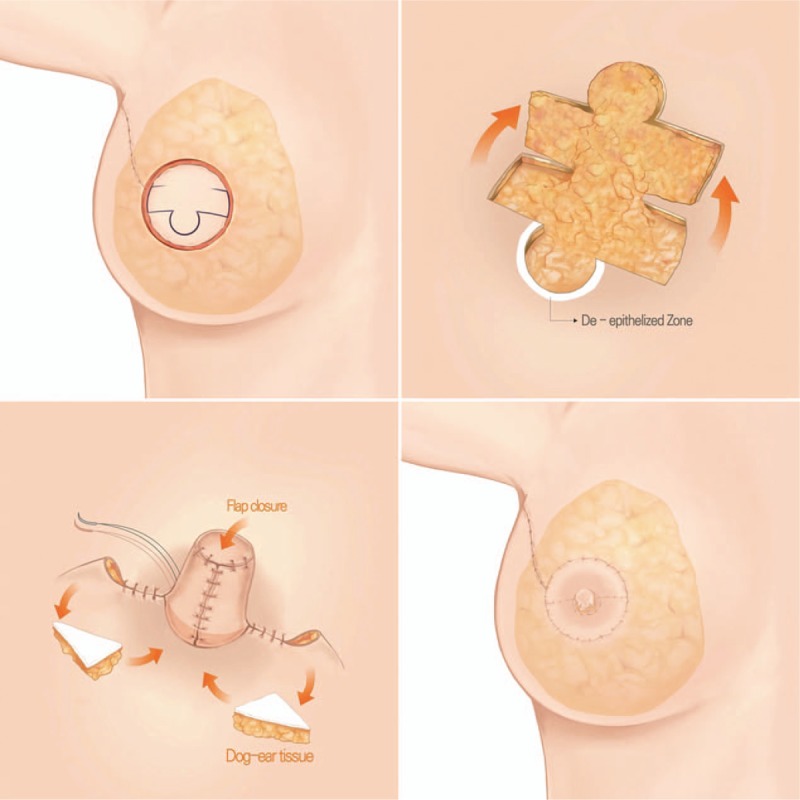
Schematic representation of the modified C-H flap for nipple reconstruction. The 4 steps of the C-H flap are as follows: design (above left), flap elevation (above right), 3) donor closure and dog ear correction (below left), and flap closure (below right). During flap elevation, a de-epithelized balcony (white crescent area) is made. De-epithelized dermofat (triangular part) is harvested from the dog ear and inserted under the base of the flap.

The modified C-H flap, which is a further-refined form of the classical C-H flap, was performed in 4 steps (Fig. 1). When designing the flap, a blunt-tip flap was preferred to a V-shaped flap. When elevating the flap, a de-epithelized crescent balcony was made in the area where the base of the reconstructed nipple would be placed. When closing the donor site, a de-epithelized dermofat tissue was harvested during dog ear correction. The dermofat tissues harvested from the dog ear were buried beneath the base of the reconstructed nipple. Finally, the reconstructed nipple was finished by wound closure with a C-H shape. In few cases, skin closure was completed 3 to 5 days later to prevent flap (C-H flap) congestion, if the flap was too edematous.

In each group, the nipple was evaluated preoperatively. After the simultaneous nipple reconstruction, the reconstructed nipple was evaluated immediately postoperatively and 1 year postoperatively. Aesthetic outcomes such as nipple projection and width and areola size were recorded. The assessment was performed with caliper by other plastic surgeon who did not perform the surgery. Data about postoperative complications of the reconstructed nipple such as delayed healing, necrosis, scar contracture, and projection loss were also recorded.

For comparison of the variables between the 2 groups, the statistical analysis was performed using the software IBM SPSS statistics version 20.0 (IBM, Armonk, NY). Values of *P ≤* .05 were considered statistically significant.

## 3 Results

In group A, the mean projection of the nipple before the surgery was 9.4 mm. The mean projection of the reconstructed nipple immediately postoperatively was 13.9 mm, whereas the mean projection of the reconstructed nipple 1 year postoperatively was 6.9 mm (Table 1). The mean projection of the reconstructed nipple decreased by approximately 50% during the postoperative 1 year.

**Table 1:**
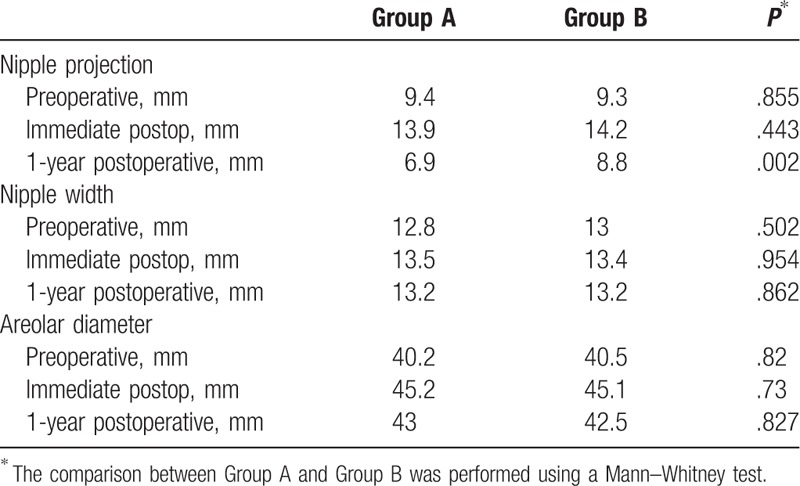
Changes in the aesthetic results of the simultaneously reconstructed nipple–areolar complex (Group A and Group B).

The mean width of the reconstructed nipple in group A was 13.5 mm immediately postoperatively and 13.2 mm 1 year postoperatively (Table 1). No significant change in the width of the reconstructed nipple was observed during the postoperative 1 year. The mean diameter of the reconstructed areola did not change much either, as the reconstructed areola was 45 mm immediately postoperatively and 43 mm 1 year postoperatively (Table 1).

In group B, the mean projection of the preoperative nipple was 9.3 mm. The mean projection of the reconstructed nipple was 14.2 mm immediately postoperatively, and 8.8 mm 1 year postoperatively (Table 1). The mean projection of the reconstructed nipple decreased by approximately 38% during the postoperative 1 year.

The mean width of the reconstructed nipple in group B did not show significant changes during the postoperative 1 year, as the reconstructed nipple width was 13.4 mm immediately postoperatively and 13.2 mm 1 year postoperatively (Table 1). Likewise, the mean diameter of the reconstructed areola did not change significantly, as the reconstructed areola was 45.1 mm immediately postoperatively and 42.5 mm 1 year postoperatively (Table 1).

Among the 51 cases, 9 showed minor complications related to the nipple–areolar complex. However, major complication such as complete necrosis of the reconstructed nipple–areolar complex was not observed in either group. In group A, there were 6 cases (26%) of minor complications, and most of them involved unsatisfactory projection of the nipple. Among them, 4 cases (17%) needed a simple revisional procedure such as redo-projection procedure or wound revision (Table 2). In group B, there were 3 cases (11%) of minor complications, and only 1 (4%) of them needed wound revision (Table 2).

**Table 2:**
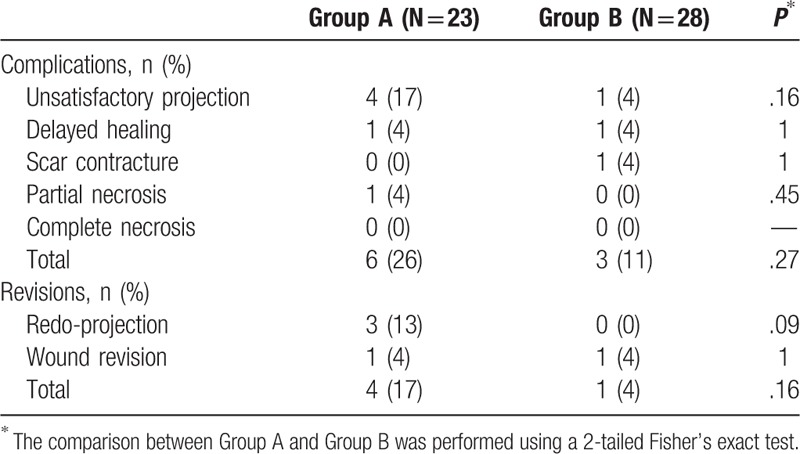
Complication rate and revision rate of the simultaneously reconstructed nipple (Group A and Group B).

## 4 Case 1

**Figure 2:**
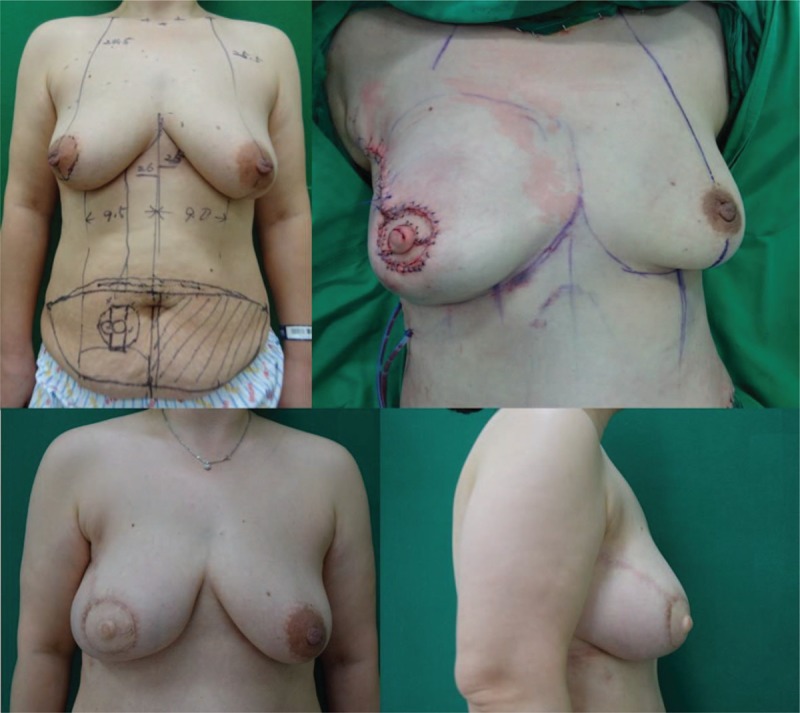
A 43-year-old female patient who underwent skin-sparing mastectomy. An immediate pedicled TRAM flap and a modified C-H flap were performed simultaneously. Preoperative (above left), immediately postoperative (above right), and 1-year postoperative (below left and right) photographs were taken. One year postoperatively, the projection of the reconstructed nipple was 8.5 mm and the width was 13.5 mm. TRAM = transverse rectus abdominis musculocutaneous.

A 43-year-old female patient underwent skin-sparing mastectomy (Fig. 2). A pedicled TRAM flap was performed to reconstruct the breast mound, and a modified C-H flap was used for the 1-stage nipple reconstruction. No complications were observed. The final height of the reconstructed nipple was similar to that of the contralateral nipple.

## 5 Case 2

**Figure 3:**
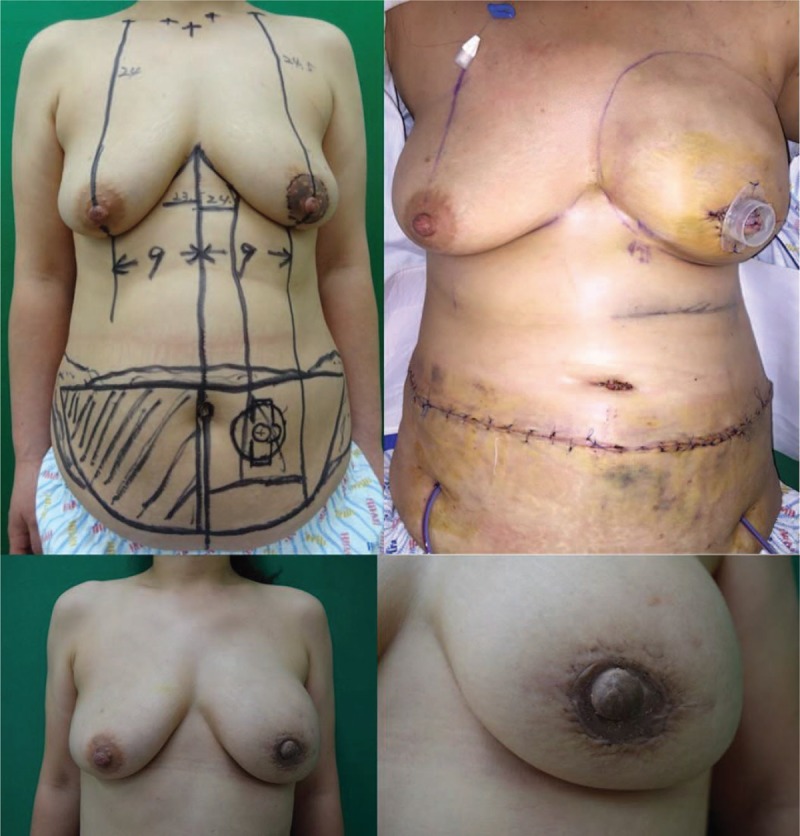
A 46-year-old female patient who underwent skin-sparing mastectomy. An immediate pedicled TRAM flap and a modified C-H flap were performed simultaneously. Preoperative (above left), immediately postoperative (above right), and 1-year postoperative (below left and right) photographs were taken. One year postoperatively, the projection of the reconstructed nipple was 10 mm and the width was 14 mm. TRAM = transverse rectus abdominis musculocutaneous.

A 46-year-old female patient underwent skin-sparing mastectomy (Fig. 3). A pedicled TRAM flap was used for the breast mound reconstruction, and the nipple was reconstructed with a modified C-H flap in 1 stage. No complications were observed. The height of the reconstructed nipple 1 year postoperatively was similar to that of the contralateral nipple.

## 6 Discussion

Simultaneous nipple–areolar complex reconstruction is a useful technique when autologous breast reconstruction is performed, as it enables reconstruction of the breast mound and nipple in 1 step.^[13–17]^ However, simultaneous reconstruction is still thought to be risky, because of the possibility of complications related to the reconstructed nipple. As the simultaneously reconstructed nipple is “a flap on a flap” in some sense, there may be circulatory problems that lead to complete or partial necrosis of the nipple. Loss of projection of the reconstructed nipple is another main problem of this procedure.^[4–10]^ Previous articles have introduced various methods of nipple reconstruction and presented their rates of projection loss.^[4–12]^ To summarize, simultaneous reconstruction of the nipple–areolar complex remains concerns about safety and projection loss. To solve these problems, many modified techniques have been introduced.^[4–8]^ However, most of them did not show significantly differentiated results.

In our cases of simultaneous nipple–areolar complex reconstruction, no major complications such as complete necrosis of the nipple were observed. Although the total complication rate was much higher in the classical C-H flap group, complications were similar between the groups except for “unsatisfactory projection.” In both groups, the rates of complications involving wound problems or flap circulation were approximately 7% to 8%. Furthermore, statistically, there was no significant difference for the complication rate between the 2 groups. We concluded that procedures in both groups were safe, and this could be obtained by establishing and carefully following the principles involved in the procedure.

The tips for ensuring the safety of the immediately reconstructed nipple begin at the flap design step. Designing the nipple–areolar complex close to the perforator is important. Detecting the perforators on the breast mound flap by hand-held Doppler and designing the nipple flap on the perforator spot ensure the survival of the reconstructed nipple. Using a C-H flap instead of a C-V flap is another tip for safety.^[18]^ The C-H flap has a broad square-shaped tip, whereas the C-V flap has a sharp, narrow V-shaped tip. As the broad tip of the flap prevents circulatory problems that can lead tip necrosis, the C-H flap is superior to the C-V flap with respect to the prevention of the tip necrosis of the flap. Delaying the skin suture on the reconstructed nipple can also be helpful. This tip was applied only in limited numbers of group B cases which showed too much edema on the reconstructed nipple. If the flap is too edematous, there can be a marginal necrosis or congestion on suture sites. In such cases, only the subdermal suture was performed at first, and a skin suture on the C-H flap was performed approximately 3–5 days after the nipple reconstruction to reduce the tension on the flap skin.

As maintaining the projection of the reconstructed nipple is an important issue in 1-stage nipple reconstruction, developing a procedure for projection maintenance is another key to success. In this study, the modified C-H flap group showed much superior results in terms of nipple projection loss. The classical C-H flap group showed approximately 50% projection loss during the postoperative 1 year, and this rate is similar to what Hammond introduced in 2007.^[19,20]^ Our modified technique reduced this projection loss rate to 38%. The projection of the reconstructed nipple of both groups at postoperative 1 year was significantly different with .002 of *P* value.

For maintaining the projection, 2 advanced procedures should be added to the classical C-H flap. The first is the crescent balcony method. When elevating the flap, a de-epithelized balcony is made with a crescent shape in the area where the base of the reconstructed nipple will be placed. This area is for supporting the reconstructed nipple. When suturing the flap, the base of the reconstructed nipple is placed on the dermal floor made by the de-epithelization. The dermal floor prevents the reconstructed nipple from sinking. The second advanced method is the transfer of the dermofat into the base of the reconstructed nipple. As the preferred design of the flap is C-H and not C-V, the dog ear always occurs at both the corners of the donor site. During the dog ear correction, the de-epithelized free dermofat can be harvested from the dog ears. When suturing the flap, the harvested free dermofat is inserted into the base of the reconstructed nipple. This also supports the base of the nipple and prevents the nipple from sinking.

In spite of performing these 2 procedures, losing the projection of the reconstructed nipple to some degree is unavoidable because of subsidence of edema with time. Although both procedures were performed, we experienced a mean projection decrease of 38% 1 year postoperatively. To overcome this decrease, the nipple reconstruction should be approximately 30% to 40% larger in projection than the opposite side nipple. The nipple that is 30% to 40% larger immediately after the operation reduces to the same size as the contralateral nipple after approximately a year.

Another factor that can affect the reconstructed nipple–areolar complex is the condition of the reconstructed breast mound. A number of factors contributing to volume shrinkage in the breast mound such as contracture following radiation therapy after surgery, and surgical complications such as fat necrosis,^[21–25]^ cause volume change in the reconstructed nipple. To overcome this aesthetic problem, we focused on improving the circulation in the autologous flap. We elevated the autologous flap with as many perforators as possible, particularly including the rectus muscle in the TRAM flap cases. Further, this procedure minimized fat necrosis by increasing not only the arterial circulation but also the venous drainage.

## 7 Conclusion

Simultaneous nipple reconstruction during breast mound reconstruction requires skillful techniques. However, the tips introduced in this paper ensure that 1-stage reconstruction is no longer a risky procedure with unfavorable results. As simultaneous reconstruction saves time and reduces costs in comparison with 2-stage reconstruction, performing simultaneous reconstruction with safe techniques is advantageous for the patients. In addition to the economic benefits, this technique provides psychological benefits, such as emotional satisfaction and relief in the immediate postoperative period. A careful surgical approach with our advanced techniques makes simultaneous breast and nipple reconstruction successful.

## Author contributions

**Conceptualization:** Jung Woo Chang, Hee Chang Ahn.

**Data curation:** Jung Soo Yoon.

**Formal analysis:** Hee Chang Ahn.

**Investigation:** Jung Soo Yoon, Jung Woo Chang.

**Methodology:** Hee Chang Ahn.

**Project administration:** Jung Soo Yoon, Jung Woo Chang, Hee Chang Ahn, Min Sung Chung.

**Resources:** Hee Chang Ahn.

**Supervision:** Jung Woo Chang, Hee Chang Ahn, Min Sung Chung.

**Writing – original draft:** Jung Soo Yoon, Jung Woo Chang.

**Writing – review & editing:** Jung Soo Yoon, Jung Woo Chang, Hee Chang Ahn, Min Sung Chung.

Jung Woo Chang: 0000-0002-7937-9679.
